# Supplementary figures and images for: Functional characterization of antennae-enriched chemosensory protein 4 in emerald ash borer, Agrilus planipennis
Source: PeerJ. 2025 Aug 18;13:e19812. doi: 10.7717/peerj.19812 (PMC12369603; doi:10.7717/peerj.19812)

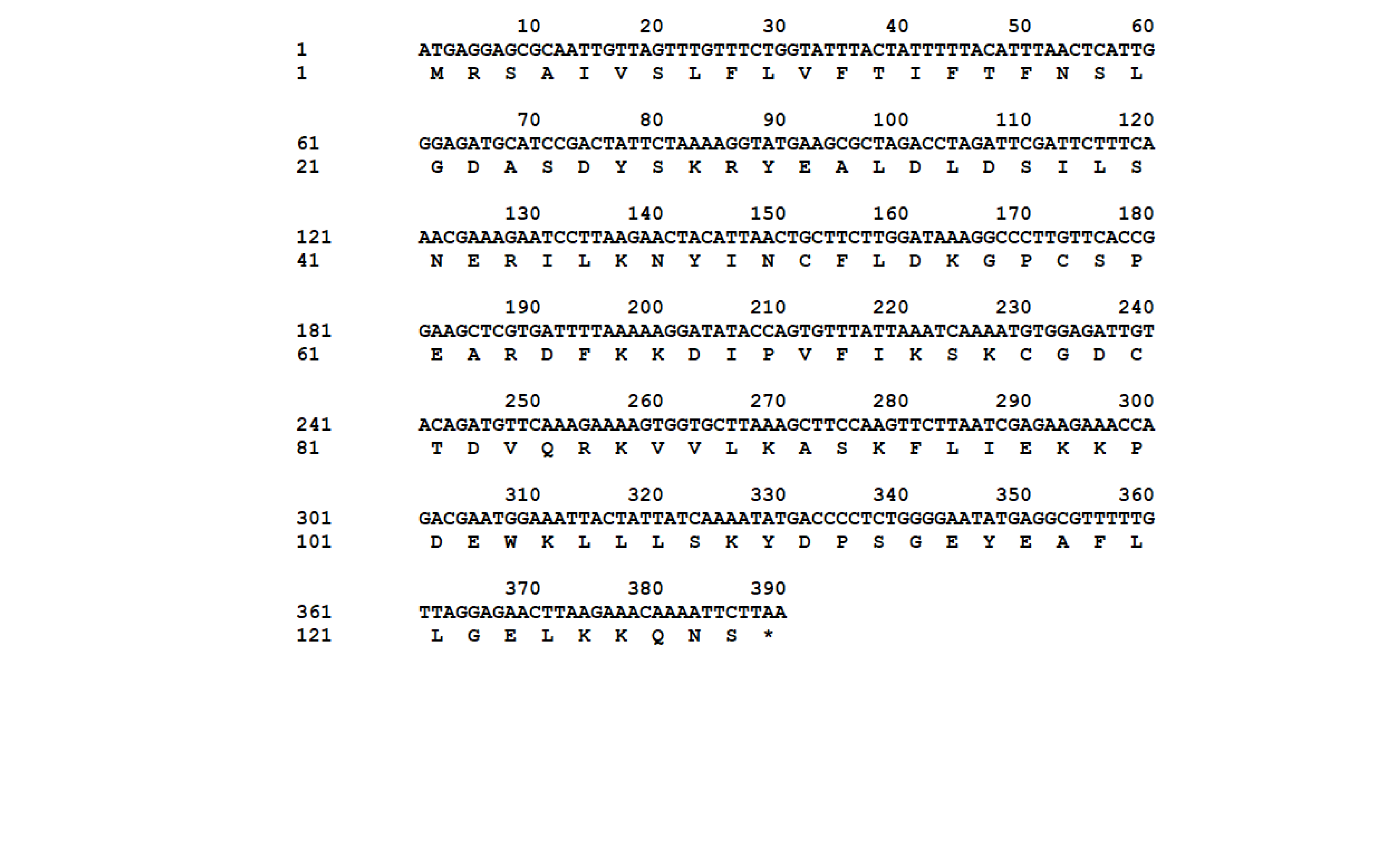

Supplement: Supplemental Information 5 [file peerj-13-19812-s005.png]

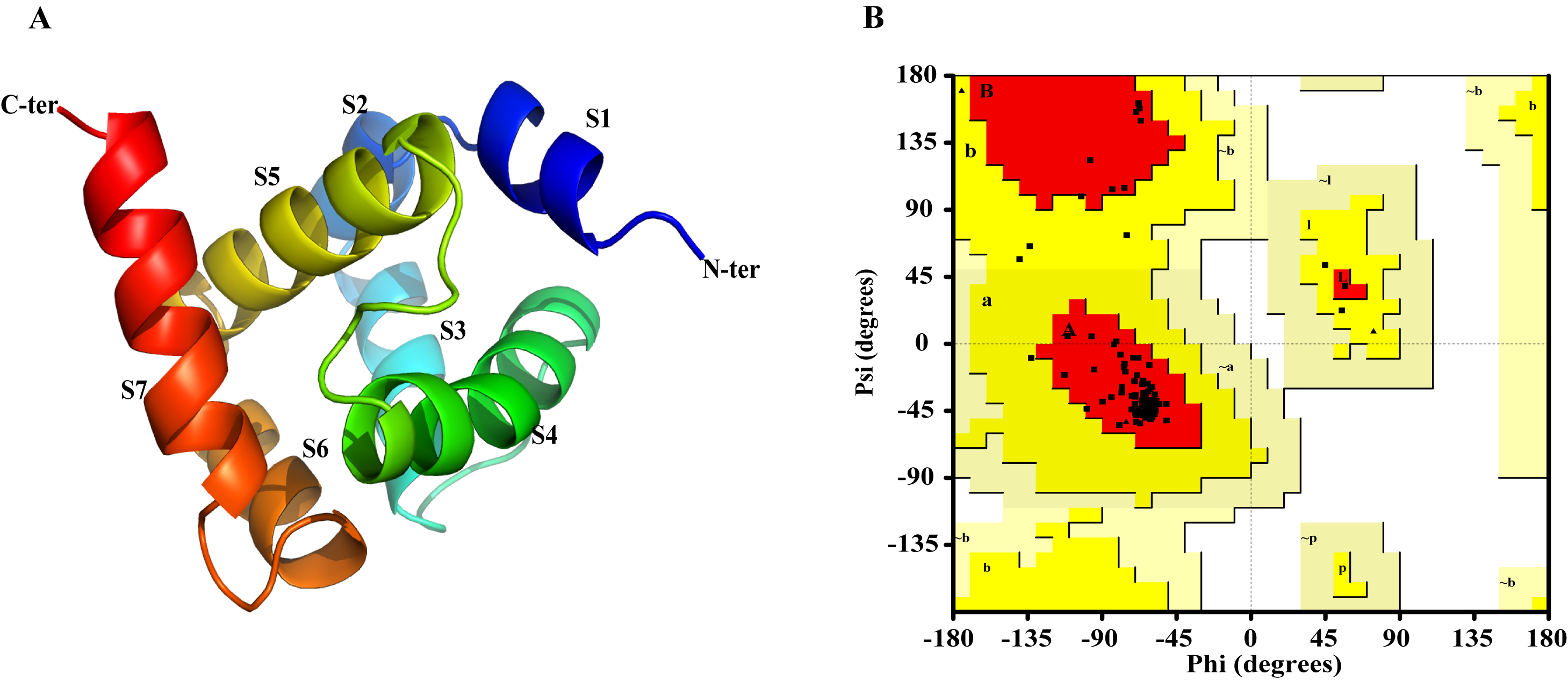

Supplement: Supplemental Information 6 — (A) is the structure of AplaCSP4, including seven helixes (S1-7). (B) indicates the ramachandran plot, and used to assess the quality of AplaCSP4. [file peerj-13-19812-s006.png]

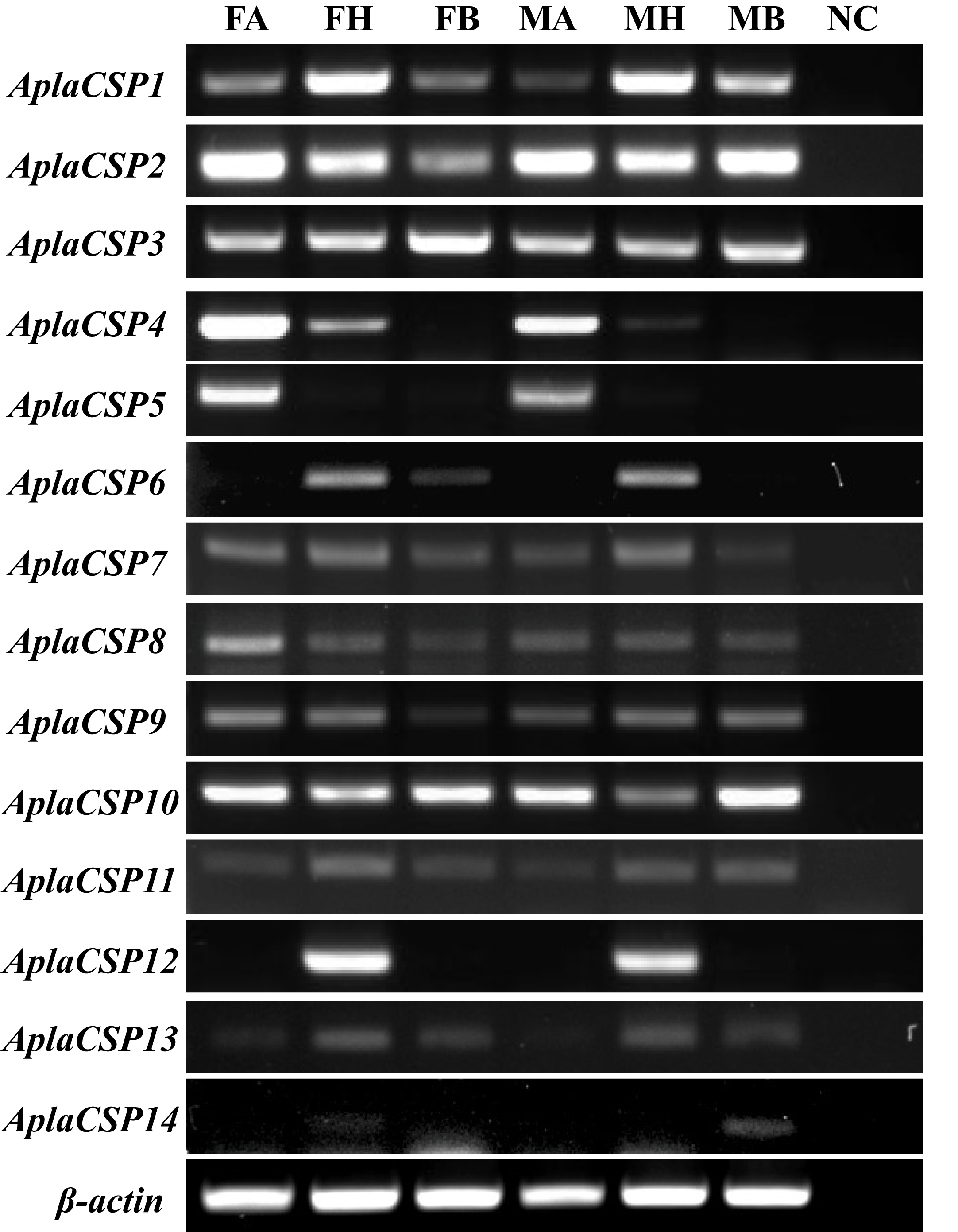

Supplement: Supplemental Information 7 — Female antennae (FA), female head (FH),female body, male antennae (MA), male head, and male body (MB) indicated the different tissues. β-actin was used as a control gene. NC was negative control. [file peerj-13-19812-s007.png]
