# Supplementary material for: Functional characterization of antennae-enriched chemosensory protein 4 in emerald ash borer, Agrilus planipennis
Source: PeerJ. 2025 Aug 18;13:e19812. doi: 10.7717/peerj.19812 (PMC12369603; doi:10.7717/peerj.19812)
Supplement: Supplemental Information 8 [file peerj-13-19812-s008.docx]

**Table S1 Primers used in this study.**

| Gene | Primer sequences | Tm (℃) | Product size (bp) | Primer use | Amplification efficiency, R^2^ |
| --- | --- | --- | --- | --- | --- |
| AplaCSP4 | F: TCCAGTAGAATTCATCCATTATG | 54.2 | 435 | Sequence verification |  |
|  | R: ACATAATCTATTTAGTTTGAATTGC | 53.0 |  |  |  |
| AplaCSP4 | F: GAGGAGCGCAATTGTTAGTTTGT | 60.7 | 307 | RT-PCR |  |
|  | R: CCATTCGTCTGGTTTCTTCTCGA | 63.2 |  |  |  |
| Actin | F: TTGGACTTCGAACAAGAAATGGC | 63.3 | 383 |  |  |
|  | R: GAAGCCAAGATAGATCCACCGAT | 61.5 |  |  |  |
| AplaCSP4 | F: AGGAGCGCAATTGTTAGTTTGT | 58.8 | 109 | qRT-PCR | 99.10%, 0.99 |
|  | R: TCGAATCTAGGTCTAGCGCTTC | 58.6 |  |  |  |
| β-actin | F: GGCATCACACCTTCTACAATGA | 57.6 | 130 |  | 98.00%, 0.99 |
|  | R: GTGTTGAAGGTTTCGAACATGA | 57.7 |  |  |  |
| EF1-α | F: CCTGGACACAGAGATTTCATCA | 57.3 | 138 |  | 97.09%, 0.99 |
|  | R: TAATGCATGTTCACGTGTTTGA | 57.6 |  |  |  |
